# Release of Neutrals in Electron-Induced Ligand Separation from MeCpPtMe_3_: Theory Meets Experiment

**DOI:** 10.1021/acs.jpca.4c08259

**Published:** 2025-02-13

**Authors:** Hlib Lyshchuk, Alexey V. Verkhovtsev, Jaroslav Kočišek, Juraj Fedor, Andrey V. Solov’yov

**Affiliations:** † J. Heyrovský Institute of Physical Chemistry, Czech Academy of Sciences, Dolejškova 3, Prague 18223, Czech Republic; ‡ Department of Physical Chemistry, University of Chemistry and Technology, Technická 5, Prague 16628, Czech Republic; § MBN Research Center, Altenhöferallee 3, Frankfurt am Main 60438, Germany

## Abstract

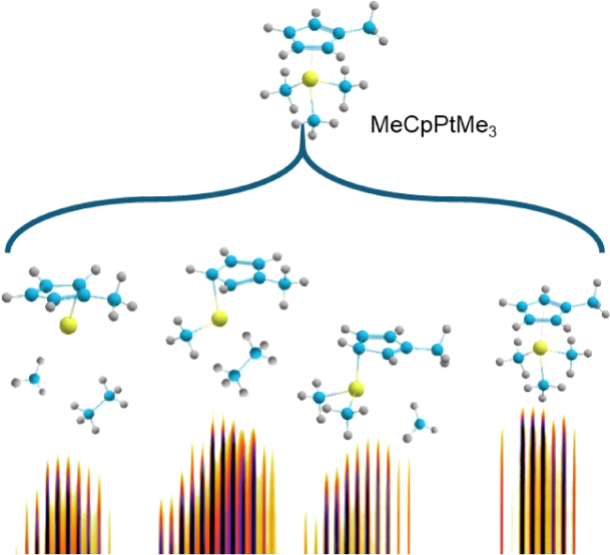

The interest in the electron impact-induced ligand release from MeCpPtMe_3_ [trimethyl(methylcyclopentadienyl)platinum(IV)] is motivated by its widespread use as a precursor in focused electron and ion beam nanofabrication. By experimentally studying the electron impact dissociative ionization of MeCpPtMe_3_ under single-collision conditions, we have found that the removal of two methyl radicals is energetically more favorable than the removal of one radical and even energetically comparable to the nondissociative ionization of MeCpPtMe_3_. This observation is explained by the structural rearrangement of the MeCpPtMe_3_^+^ ion prior to dissociation, resulting in the removal of ethane instead of two methyl groups. This fragmentation pathway is computationally confirmed and studied by irradiation-driven molecular dynamics (IDMD) simulations. The formation of complex molecules in irradiation-induced molecular dissociation is a general phenomenon that can occur in various molecular systems. This study explains the puzzling results of previous experiments with MeCpPtMe_3_ molecules and highlights the use of the IDMD approach to describe radiation-induced chemical transformations in molecular systems.

## Introduction

Focused electron and ion beam nanofabrication techniques^1−3^ exploit the spatially localized irradiation-induced chemistry with organometallic precursor molecules deposited on a surface.^4,5^ The collimated beams of charged particles induce chemical reactions of adsorbed molecules, resulting in the formation of a deposit on the surface, thus enabling direct-write 3D nanoprinting of complex nanostructures.^6,7^ A commonly used compound for the fabrication of platinum-containing deposits in commercial gas-injection systems is MeCpPtMe_3_ or (CH_3_C_5_H_4_)Pt(CH_3_)_3_.^5,8^ The main reason for this is the exceptional thermal stability of the MeCpPtMe_3_ precursor, which can be used for years without refilling the gas-injection system. However, this fact is counterbalanced by the low purity of the as-grown deposits, where the platinum content typically does not exceed 15–17%,^5,8^ thus requiring the use of various purification techniques.^5,9,10^

The low metal content of the Pt-containing deposits is surprising, given the performance of MeCpPtMe_3_ in chemical vapor deposition, where 99% platinum content can be achieved.^8^ The differences in the composition of the nanostructures grown by different deposition techniques have stimulated interest in studying the mechanisms of electron-induced fragmentation of MeCpPtMe_3_. The primary question is how many (and which) ligands are removed from this molecule in collisions with electrons. While the energy of the primary beams is typically in the keV range, the main focus has been on the electron energies in the 0–100 eV range, since the radiation-induced chemistry during the deposition process is largely influenced by the low-energy secondary electrons^3^ present in the interaction region in both focused electron- and ion-beam deposition techniques (FEBID and FIBID).^3,11^ Electron-induced fragmentation of MeCpPtMe_3_ has been studied both in the gas phase^12^ and in the condensed phase.^10,13^ In the latter approach, volatile fragments released upon electron irradiation of condensed MeCpPtMe_3_ layers were monitored. This gave puzzling results, as no methyl (CH_3_) fragments were reported in both studies.^10,13^ The dominant species released was methane (CH_4_), with a small contribution of ethane (C_2_H_6_). The lack of methyl radical release was tentatively attributed to their reactive trapping in the condensed layers.

Inspired by these puzzles, this joint experimental and computational study addresses the question of how the MeCpPtMe_3_ fragmentation pattern (ligand loss) depends on the electron energy. Electron-induced fragmentation can proceed via dissociative ionization (DI; positive ion fragmentation pathway), dissociative electron attachment (DEA; negative ion fragmentation pathway), or neutral dissociation.^1^ Energy-resolved data for MeCpPtMe_3_ are available in the literature for DEA,^12^ whereas for DI, only the mass spectrum at 100 eV electron energy has been reported.^12^ In this study, we consider the DI of MeCpPtMe_3_ due to the collision with low-energy electrons (with energy up to 20 eV) and demonstrate that the removal of two methyl radicals is energetically more favorable than the removal of one radical and even energetically comparable to the nondissociative ionization of MeCpPtMe_3_. This observation is explained, using advanced atomistic simulations and quantum chemistry calculations, by the structural rearrangement of the MeCpPtMe_3_^+^ ion prior to dissociation, resulting in the removal of ethane instead of two methyl groups.

## Experimental Methods

The experimental results were obtained using the Cluster Beam (CLUB) setup.^14,15^ Isolated MeCpPtMe_3_ molecules were studied by introducing them into the time-of-flight chamber as a background gas, similar to our previous studies.^16−18^

The MeCpPtMe_3_ sample (purchased from Strem Chemicals, Inc.) was introduced into the interaction chamber and exposed to low-energy electrons produced by a magnetically collimated electron gun with a heated tungsten filament. The electron energy can be varied between 0 and 80 eV. The base pressure in the chamber is 1 × 10^–8^ mbar. During the measurements, the background pressure of the sample reached ∼4 × 10^–7^ mbar, ensuring the single-collision conditions of the experiment. The product ions were extracted from the interaction region into a reflectron time-of-flight mass spectrometer. The mass resolution of the spectrometer is *M*/Δ*M =* 4000.

The experimental appearance energy *E*_A_ for a given ion fragment was determined by fitting the dependence of the ion yield on the electron energy *E*_*e*_. The ion yield was obtained by integrating the signal intensity over the entire selected mass peak. The fitting procedure used the generalized Wannier law,^19^ described by the function:
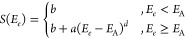
1where the background *b*, the multiplication factor *a*, the Wannier exponent *d*, and *E*_A_ are fitting parameters. Given the energy resolution of our electron gun (approximately 600 meV), the following approach was used. The parameter *b* was determined in the energy range below *E*_A_, and the local minima fits with different initial values of *E*_A_, *b*, and *d* were performed using the Levenberg–Marquardt algorithm as implemented in Gnuplot.^20^ The results are summarized in Table S1. The electron energy was calibrated for the appearance energies of ions from the background molecules O_2_ and N_2_ against their ionization energies published by NIST.

## Computational Methods

The physical origin of the experimentally measured fragmentation pattern has been elucidated by irradiation-driven molecular dynamics (IDMD) simulations of MeCpPtMe_3_^+^ fragmentation and quantum chemical calculations using density functional theory (DFT). Details of the used computational procedure are given below in this section.

### IDMD Simulations

IDMD simulations of radiation-induced fragmentation of MeCpPtMe_3_^+^ were performed using MBN Explorer,^21^ a software package for multiscale simulations of the structure and dynamics of complex meso-bionano (MBN) systems.^22^ The MBN Studio toolkit^23^ was used to create the system’s structure, prepare the necessary input files, and analyze the simulation results.

The IDMD method^24^ allows atomistic simulations of irradiation-driven transformations in various molecular and condensed matter systems.^24−31^ This method was introduced in ref (24) and described in detail in several recent reviews and books.^25−27^ The key ideas of this method are briefly summarized below.

Within the IDMD framework, various quantum processes occurring in a system exposed to irradiation (e.g., covalent bond breakage induced by ionization or electron attachment) are treated as random, fast and local transformations incorporated into the classical MD framework in a stochastic manner.^24^ The probability of each quantum process is equal to the product of the process cross section (which can be obtained from *ab initio* calculations, analytical models, or taken from experiments) and the flux density of incident particles.^32^ Transformations of irradiated molecular systems (e.g., molecular topology changes, redistribution of atomic partial charges, or alteration of interatomic interactions) are simulated by means of MD with the reactive CHARMM (rCHARMM) force field^33^ described in the next subsection.

The IDMD methodology allows the analysis of fast energy transfer events into fragmenting covalent bonds caused by the above-mentioned quantum processes, as well as the analysis of postirradiation energy relaxation processes that typically occur on the picosecond time scale and lead to chemical transformations.^24,26,27,34,35^ In the fast and localized energy transfer mechanism, the energy remaining in the system after ionization (i.e., the excess energy over the ionization energy of the parent molecule) is locally transferred to a specific covalent bond of the parent ion and converted into the kinetic energy of the two atoms forming the bond. In the slow thermal energy transfer mechanism, the energy stored in the electronic degrees of freedom is statistically distributed to the vibrational degrees of freedom of the parent ion. The equilibrium atomic velocities corresponding to a given temperature are scaled depending on the amount of energy transferred to the system.

Within the IDMD-based model of molecular fragmentation considered in this study it is assumed that the energy transferred to the parent molecular system by irradiation is transferred from the electronic degrees of freedom of the system to its vibrational degrees of freedom by the beginning of the simulation, and the evolution of the system is simulated classically using the reactive rCHARMM force field.^33^ The parameters for the bonded and angular interactions for different covalent bonds in MeCpPtMe_3_^+^ and molecular fragments change dynamically upon the breakage or formation of covalent bonds (see below and the Supporting Information).

### rCHARMM Force Field

The interatomic interactions for MeCpPtMe_3_^+^ and its fragments have been described using the rCHARMM force field.^33^ It allows the simulation of various molecular systems with the dynamically changing molecular topology,^28−31,34−38^ which is essential for modeling chemical transformations in various molecular and condensed matter systems, including those induced by irradiation.^26^

To permit the rupture and formation of covalent bonds, the radial part of the bonded interactions is described in rCHARMM by means of the Morse potential:^33^
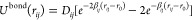
2where *D*_*ij*_ is the dissociation energy of the bond between atoms *i* and *j*, *r*_0_ is the equilibrium bond length, and the parameter  (with  being the bond force constant) determines the steepness of the potential. The bonded interactions are truncated at a user-defined cutoff distance beyond which the covalent bond is broken and the molecular topology of the system changes.

The rupture of covalent bonds in the course of simulation automatically employs a modification of the potential functions for valence angles:^33^
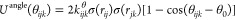
3where θ_0_ is the equilibrium angle formed by a triplet of atoms *i*, *j* and *k*; *k*^θ^ is the angle force constant; and the sigmoid-type function σ(*r*_*ij*_) describes the effect of bond breakage.^33^

Nonbonded van der Waals interactions between atoms of the system have been described by means of the Lennard-Jones potential:
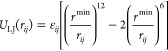
4where  and . The electrostatic interaction between atoms with nonzero partial charges have been described using the Coulomb potential.

The rCHARMM parameters for the bonded and angular interactions, nonbonded interactions, and the atomic partial charges in the parent MeCpPtMe_3_^+^ ion and the created molecular fragments are listed in Tables S2–S5.

### DFT Calculations

Density functional theory (DFT) calculations were performed with Gaussian 16 software.^39^ The calculations employed the hybrid B3LYP functional^40^ with the LanL2DZ^41^ basis set and the GD3 empirical dispersion correction.^42^

The first set of calculations was performed to determine the rCHARMM force field parameters for IDMD simulations of MeCpPtMe_3_^+^ fragmentation. A series of relaxed potential energy scans were performed for different covalent bonds in the parent MeCpPtMe_3_^+^ ion and for Pt–C bonds in the MeCpPtMe_2_^+^ and MeCpPtMe^+^ fragments. For each reaction coordinate, the interatomic distance was varied in steps of 0.1 Å, and the geometry optimization calculation was performed for each specific value of the interatomic distance. Similar complementary calculations were performed for the C–C and C–H bonds in a neutral C_2_H_6_ molecule. The calculated potential energy curves were fitted with the Morse potential, eq 2, to determine the bond dissociation energies and force constants. The corresponding parameters are listed in Table S2.

The second set of calculations was performed to determine the most probable reaction channels by calculating the appearance energies of the fragments for a given channel. Geometry optimization calculations were performed for MeCpPtMe_3–*n*_^+^ (*n* = 0–3) species and [MeCpPtMe_3–*n*_^+^ – *m*H] (*n* = 0–3) fragments corresponding to the loss of *m*H atoms, as well as for possible neutral fragments C_2_H_6_, CH_4_, CH_3_, H, and H_2_.

The third set of calculations was performed to explore the potential energy surface (PES) for the MeCpPtMe_3_^+^ ion. An extensive series of relaxed energy scans (1140 independent calculations) were performed for different geometric configurations of MeCpPtMe_3_^+^, enabling to identify a transition state separating the two energetically favorable configurations (the optimized geometry of MeCpPtMe_3_^+^ and the end point configuration corresponding to the formation of an ethane molecule) described in the Results and Discussion section.

### Protocol for the Simulation of MeCpPtMe_3_^+^ Fragmentation

The methodology adopted in this study for simulating the fragmentation of MeCpPtMe_3_^+^ is based on the methodology previously used to simulate radiation-induced fragmentation of other organometallic molecules.^34,35^ First, the geometry of a MeCpPtMe_3_^+^ cation was optimized with MBN Explorer using the rCHARMM force field and the parameters listed in Tables S2–S5. The system was then thermalized at *T* = 300 K for 100 ps. The constant-temperature simulations were performed using the Langevin thermostat with a damping time of 0.2 ps. Ten independent MD simulations were performed for statistical purposes. In each simulated trajectory, atomic coordinates and velocities were recorded every 10 ps. The trajectories were used to generate initial geometries and velocity distributions for the IDMD simulation of the fragmentation process.

The IDMD simulations of radiation-induced MeCpPtMe_3_^+^ fragmentation were performed in a large simulation box with a side length of 800 Å. The simulations were run for 0.5 ns with an integration time step of 0.1 fs without a thermostat. 1560 simulations were performed for different excess energy values ranging from 0 to ∼10.8 eV (250 kcal/mol) in steps of ∼0.43 eV (10 kcal/mol). For each energy, 60 independent simulations were performed. The molecular fragments produced at the end of the simulations were analyzed, and the corresponding fragment appearance energies and their abundances were evaluated from this analysis.

## Results and Discussion

### Fragmentation Pattern as a Function of Electron Energy

**Figure 1:**
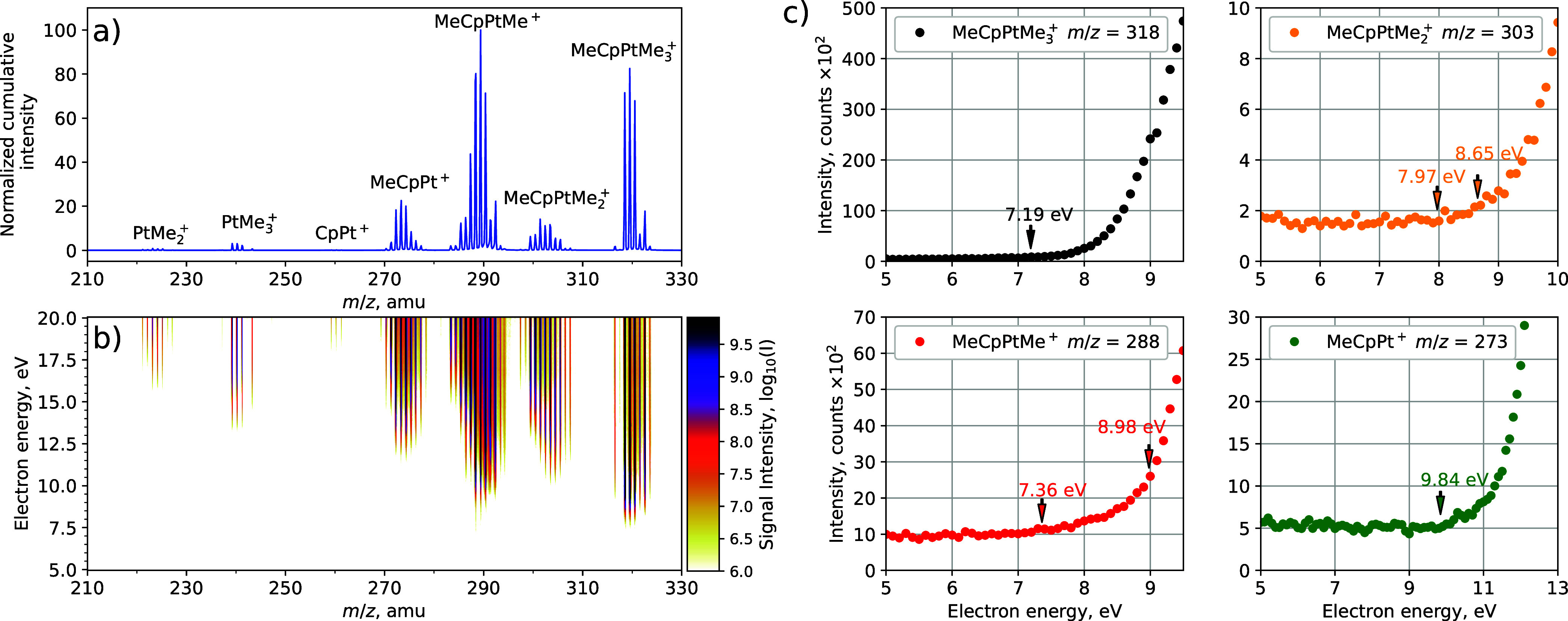
(a) Cumulative normalized fragmentation mass spectrum of MeCpPtMe_3_ due to collisions with 5–20 eV electrons for the fragment mass range of 210–330 amu; (b) mass spectrum as a function of electron energy (*y*-axis) with intensity color-coded; (c) ion yield as a function of electron energy for four selected mass peaks. The data in each subpanel correspond to the given mass peak, the chemical assignment has been made assuming a ^194^Pt isotope. The vertical arrows indicate the threshold energies calculated with DFT (see Table S1).

The experimental results on the electron impact induced DI of MeCpPtMe_3_ are shown in Figure 1. Panel (a) shows the cumulative mass spectrum (i.e., the sum of all the mass spectra taken with the energy step of 0.1 eV) in the electron energy range of 5–20 eV. The spectrum is rich due to five major isotopes of platinum (*m* = 192, 194, 195, 196, and 198 amu) and a different number of H atoms that could be cleaved from each fragment. Nevertheless, the main groups of fragments can be easily identified. The group of peaks in the range *m*/*z* = 316–324 corresponds to the parent MeCpPtMe_3_^+^ ion. Three groups of peaks in the range *m*/*z* = 270–305 correspond to the formation of MeCpPtMe_3–*n*_^+^ (*n* = 1–3) fragments in which one, two and three methyl ligands have been removed from the parent ion, respectively.

It should be noted that each mass peak in each group of fragments may contain contributions from different platinum isotopes with different numbers of H atoms removed. For example, the peak at *m*/*z* = 303 may be due to the loss of: (i) 13 amu fragment(s) from MeCp^192^PtMe_3_^+^ (316 amu) (ii) 15 amu fragment(s) from MeCp^194^PtMe_3_^+^ (318 amu), (iii) 16 amu fragment(s) from MeCp^195^PtMe_3_^+^ (319 amu), (iv) 17 amu fragment(s) from MeCp^196^PtMe_3_^+^ (320 amu), or (v) 19 amu fragment(s) from MeCp^198^PtMe_3_^+^ (322 amu). We have assumed a natural abundance of platinum and carbon isotopes and performed a least-squares fit of the contributions of different possibilities (i.e., different numbers of cleaved H atoms) to each mass peak. Details of the fitting procedure are given in the Supporting Information, and the results are shown in Table S1.

Figure 1b shows the variation of the mass spectrum with the electron energy, with the peak intensity being color-coded. A general trend in such 2D ionization maps is that the mass peaks corresponding to a higher degree of precursor fragmentation have higher appearance energies (AEs) since the cleavage of a larger number of covalent bonds is energetically more demanding.^16^ In contrast, Figure 1b shows a different trend: the AE for the MeCpPtMe^+^ fragment (corresponding to the loss of two methyl ligands) is lower than that for the MeCpPtMe_2_^+^ fragment produced by the release of a single ligand.

Figure 1c shows the ion yield for one peak from each group of fragments, together with the threshold energies calculated using DFT (see Table S1). The calculated adiabatic ionization energy for MeCpPtMe_3_ (7.19 eV) is in good agreement with the onset of the ion yield curve for the *m*/*z* = 318 peak, corresponding to the parent ion MeCpPtMe_3_^+^. For the peak *m*/*z* = 303, we show two thresholds: the higher one at 8.65 eV corresponds to the simple bond-cleavage channel MeCpPtMe_2_^+^ + CH_3_. However, an experimental signal appears clearly below this value, and its onset agrees well with the calculated threshold of 7.97 eV, corresponding to the channel where CH_4_ is formed (the leaving methyl group abstracts an additional H atom). The ion yield curve for the peak *m*/*z* = 288 shows a low onset, its position being in good agreement with the calculated threshold for the MeCpPtMe^+^ + C_2_H_6_ channel (7.36 eV). The second lowest threshold (8.98 eV), corresponding to the formation of one methyl and one methane fragment, is significantly higher than the experimental onset. Finally, the ion yield curve for *m*/*z* = 273 again shows a relatively high onset in good agreement with the calculated threshold for the MeCpPt^+^ + C_2_H_6_ + CH_3_ channel (9.84 eV). There is also a hypothetical channel in which methane and ethane are produced as neutral fragments (8.22 eV threshold); however, no signal is visible around this threshold, so we have not marked its position in Figure 1c to avoid confusion. Interestingly, this channel becomes quite strong at higher electron energies (see Table S1). Its absence at the energy threshold could be due to a barrier in this complex rearrangement channel.

### Atomistic Details of Fragmentation Dynamics

The physical origin of the fragmentation pattern presented in the previous section, in particular the low onset for the removal of two ligands, has been elucidated by IDMD simulations of MeCpPtMe_3_^+^ fragmentation and DFT calculations.

First, the bond dissociation energies (BDE) for the Pt–C bonds in the parent MeCpPtMe_3_^+^ ion and its fragments have been determined from potential energy scans calculated by DFT (see Table S2). These calculations were preceded by the geometry optimization of a neutral MeCpPtMe_3_ molecule, followed by the optimization of the MeCpPtMe_3_^+^ cation. According to the performed calculations, in the optimized geometry of a neutral MeCpPtMe_3_ molecule, the Pt atom is positioned equidistantly to the carbon atoms of the methyl ligands. In contrast, the optimized geometry of the MeCpPtMe_3_^+^ cation is characterized by the asymmetry in the Pt–C bonds (the bond lengths are listed in Table S2). Accordingly, the Pt–C bond dissociation energy for two methyl groups in MeCpPtMe_3_^+^ is equal to 1.52 eV, while the third methyl group is more strongly bound to the Pt atom with a BDE of 2.50 eV. After the loss of one methyl group, the remaining CH_3_ ligands in the MeCpPtMe_2_^+^ fragment are strongly bound to the metal atom, and the Pt–C BDE increases to 3.21 eV. Finally, after the loss of two methyl groups, the BDE for the remaining Pt–C bond in the MeCpPtMe^+^ fragment is equal to 2.73 eV. This information has been used to develop a chemically accurate fragmentation model for IDMD simulations, allowing the change of atom types and corresponding simulation parameters (such as equilibrium bond lengths and dissociation energies) based on the information derived from the DFT calculations.

**Figure 2:**
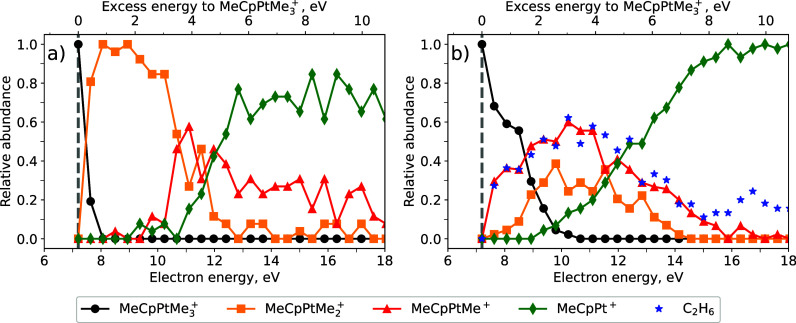
Relative abundances of the parent MeCpPtMe_3_^+^ ion and MeCpPtMe_*n*_^+^ (*n* = 0–2) fragments as a function of the electron energy (lower *x*-axis) and the energy transferred to the parent cation (upper *x*-axis), calculated using IDMD. (a) Simulations accounting for the changes in atom types and the corresponding interaction parameters upon the formation of a given fragment (see the main text for details). (b) Simulations for the fragmentation pathway corresponding to the formation of ethane. The vertical dashed lines indicate the adiabatic ionization energy of the neutral parent molecule MeCpPtMe_3_ as determined by the DFT calculations.

Figure 2a shows the abundance of MeCpPtMe_3–*n*_^+^ (*n* = 0–3) ionic species calculated by means of IDMD, corresponding to the thermal mechanism of energy transfer and taking into account the changes in the Pt–C bonded interactions described above. The *x*-axes correspond to the kinetic energy of the incident electrons (lower axis) and the excess energy given to the singly charged parent ion MeCpPtMe_3_^+^ (upper axis). The vertical dashed line indicates the adiabatic ionization energy of the parent neutral molecule, 7.19 eV, as determined by the DFT calculations. The abundance profiles shown in Figure 2a describe a “typical” behavior where the number of released ligands increases monotonically with increasing the excess energy given to the system. Similar behavior has also been observed in the localized energy transfer mechanism when the energy is transferred locally to a specific covalent bond (see Figure S2). In this case, one methyl group is rapidly ejected from the parent ion by cleavage of one of the weak Pt–C bonds. If the energy transferred to the system exceeds the BDE for the particular bond, the remaining energy is redistributed over the formed molecular fragment, resulting in the removal of one of two additional methyl groups. Such a sequential release of ligands is common for metal–carbonyl precursors,^16,34,35,43^ but is clearly not the case for MeCpPtMe_3_, as shown experimentally here (see Figure 1).

**Figure 3:**
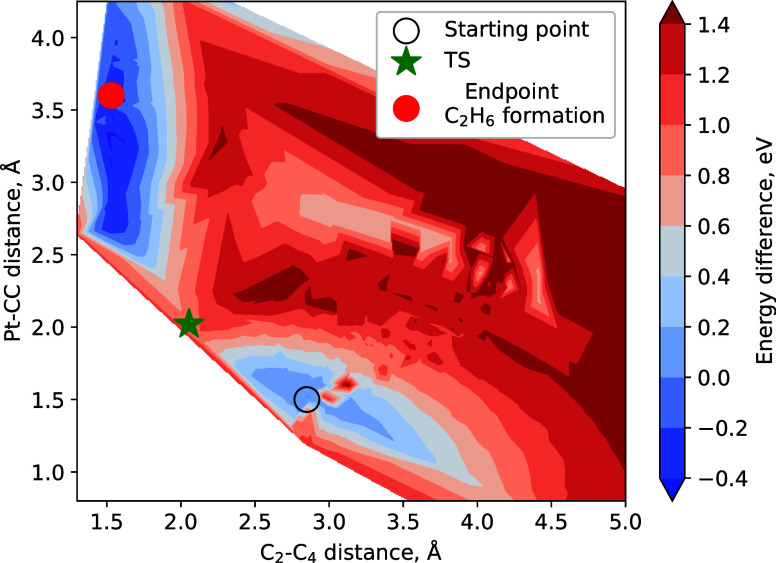
Contour plot of the potential energy surface representing different geometric configurations of the MeCpPtMe_3_^+^ ion as obtained at the B3LYP/LanL2DZ level of theory. The *x*-axis shows the distance between the C_2_ and C_4_ atoms in the two methyl ligands, which are weaker bound to the Pt atom (see Figure 4). The *y*-axis shows the distance between the Pt atom and the center of mass of the C_2_ and C_4_ atom pair. The energy (color-coded) is relative to the optimized geometry of the parent MeCpPtMe_3_^+^ ion. The symbols indicate the starting point corresponding to the optimized geometry of MeCpPtMe_3_^+^, the end point where a neutral ethane molecule is formed, and the transition state (TS) separating these two configurations.

**Figure 4:**
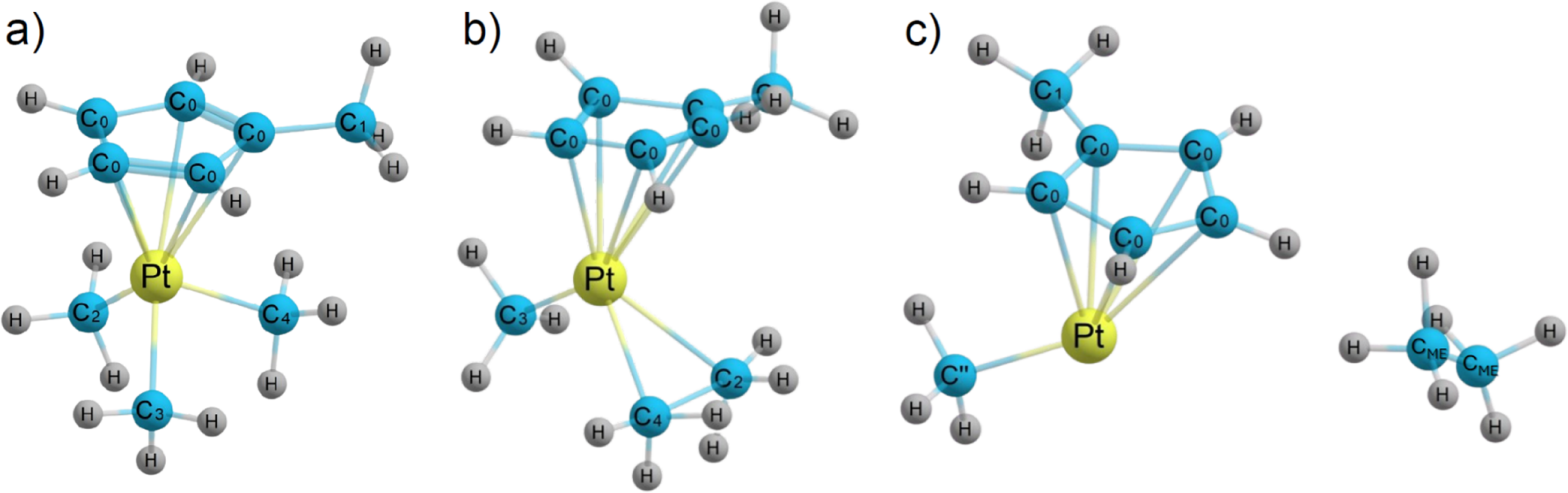
(a) Optimized geometry of the MeCpPtMe_3_^+^ ion used for the IDMD simulations with different atom types indicated. Snapshots of the IDMD simulations showing the formation of a transient complex when the carbon atoms of two methyl ligands come close to each other (panel (b)) and the moment when the ethane molecule is formed by the cleavage of two Pt–C bonds (panel (c)). The atom types are changed in the simulation during this transformation (see the Supporting Information). The simulation snapshot (c) is taken 0.4 ps after the snapshot (b).

In order to explain the unusual ordering of appearance energies, additional DFT calculations have been performed to explore the potential energy surface (PES) for the MeCpPtMe_3_^+^ ion. The contour plot of the PES is shown in Figure 3. The *x*-axis represents the distance between the C_2_ and C_4_ atoms in the two methyl ligands, which are less strongly bound to the Pt atom (see atomic notations in Figure 4), and the *y*-axis represents the distance between the Pt atom and the center of mass of the C_2_ and C_4_ atoms. The colors indicate the energy difference between each particular geometric configuration of MeCpPtMe_3_^+^ and its optimized geometry.

The PES shown in Figure 3 has two distinct minima. One energy minimum corresponds to the optimized geometry (see the open black circle), while the other corresponds to the configuration involving the formation of an ethane molecule, where the C_2_–C_4_ bond is equal to ∼1.53 Å (see the red circle). The latter configuration is ∼0.4 eV lower in energy than the optimized geometry of MeCpPtMe_3_^+^. This reaction pathway proceeds through a transition state (TS; see the green star in Figure 3) with an energy barrier of ∼0.92 eV, which is lower than the energy of ∼1.45 eV required for the loss of a single methyl group. Therefore, the fragmentation pathway leading to the formation of ethane is energetically more favorable than the removal of one methyl radical.

As an initial guess for the TS identification, we have chosen a configuration with the highest energy along the possible reaction path from the optimized configuration to the end point configuration with ethane formation. In the TS, the Pt–C_2_ and Pt–C_4_ interatomic distances are equal to ∼2.3 Å, which is only ∼0.2 Å larger than the equilibrium Pt–C_2_ and Pt–C_4_ bond lengths. As the carbon atoms of the two methyl ligands approach each other, a transient complex is formed, which decays by the simultaneous cleavage of two Pt–C bonds and the formation of a neutral ethane molecule.

The outcomes of the PES analysis using DFT have been used to extend an IDMD-based fragmentation model allowing the formation of a transient complex through the formation of a C–C bond between C_2_ and C_4_ atoms (see Figure 4b and the Supporting Information). The simulations also took into account the changes in atom types and the redistribution of atomic partial charges upon cleavage of covalent bonds (see Table S5). The results of the corresponding IDMD simulations are shown in Figure 2b. The stars indicate the formation of ethane, showing that at electron energies below ∼12 eV (excess energy below ∼5 eV), the most probable fragmentation pathway is the formation of MeCpPtMe^+^ and a neutral ethane molecule (see the simulation snapshot in Figure 4c). The single methyl loss pathway occurs at higher electron energies when the energy transferred to the system is sufficient to overcome the barrier for Pt–C bond dissociation. For electron energies above ∼12 eV, the main fragment produced in the IDMD simulations is MeCpPt^+^, which is formed either by the removal of three methyl radicals from the parent ion or by the formation of ethane and a methyl radical. As follows from the performed simulations, the characteristic time scale for the cleavage of Pt–C bonds and the formation of ethane is in the range from a few tens to a few hundreds of picoseconds. At lower values of the excess energy given to the MeCpPtMe_3_^+^ parent ion (below approximately 2 eV), the fragmentation is usually delayed, while it happens more rapidly at higher values of the excess energy.

### Relevance to Previous Studies

The performed IDMD simulations of MeCpPtMe_3_^+^ fragmentation and the corresponding DFT calculations thus explain the experimentally observed ordering of the AEs for fragment ions (Figure 1b). The results of the present study also partially explain the results of the surface science studies.^10,13^ There, however, ethane was a minor product released after the irradiation of condensed MeCpPtMe_3_ layers. The major product detected was methane (CH_4_), and no methyl radicals were detected. Experimentally, we also provide evidence for methane production under single-collision conditions (the ion yield curve of *m*/*z* = 303 in Figure 1c shows an onset at the 7.97 eV threshold). This indicates a high reactivity of the methyl radicals. In surface-based studies, where the molecules are aligned in monolayers, the dominance of the methane formation channel can most likely be attributed to ultrafast hydrogen abstraction from the neighboring molecules by the methyl radical.

This study demonstrates that the formation of complex organic molecules is due to structural rearrangement in the ionic state prior to dissociation of MeCpPtMe_3_. This process is of a general nature. Indeed, ion rearrangement prior to dissociation has been hypothesized for singly charged amino acids,^44^ plasma-enhanced chemical vapor deposition precursors,^45^ and doubly charged adamantane,^46^ as well as for anions of molecular radiosensitizers.^47^ In this context, it is interesting to note an earlier study^48^ on the dissociative ionization of deoxyribose (C_5_H_10_O_4_), where the C_5_H_6_O_2_^+^ fragment was reported to have an AE even lower than that of the parent ion. This was attributed^48^ to either the thermal decomposition of the sample or the presence of a linear isomeric structure of deoxyribose in the sample. The results of our present study allow for an alternative interpretation of that earlier finding. Since the C_5_H_6_O_2_^+^ fragment can be produced by the formation of two H_2_O molecules as neutral cofragments in the DI of deoxyribose, its observed low AE could have been caused by the ion-rearrangement similar to that described here for MeCpPtMe_3_.

It should be noted that no atomistic description of such processes has been performed so far. Here, we present the first IDMD-based study, using the reactive rCHARMM force field, of the complex irradiation-driven chemical transformation accompanied by the formation of new molecules. The utilized methodology allows one to include in the computational framework many more chemical transformations than those considered in this study. It has already been used for atomistic simulations of radiation-induced chemistry during the FEBID process^24,28−31^ and can be applied to many other systems relevant to plasma physics and technology, atmospheric and astrochemistry, or the interaction of ionizing radiation with living tissue.^26^

Besides rCHARMM, another popular reactive force field is ReaxFF,^49^ which has been parametrized and used for a large number of different systems and processes.^50^ Although ReaxFF parameter sets exist for many elements of the periodic table, the ReaxFF considers only a single atom type for each element. Therefore, to simulate bond-breaking and formation processes, ReaxFF is constructed as a relatively complex force field with many parameters.^51^ This inherent limitation of ReaxFF does not allow simulations of the reaction pathways considered in this study, which require changing the atom types for atoms of the same element.

## Conclusion

In conclusion, the experiments described in this study show that ligand separation from MeCpPtMe_3_ in the process of electron impact-induced dissociative ionization proceeds by the removal of two methyl ligands, which has a lower energetic threshold than the removal of one ligand. Quantum chemical analysis of a potential energy surface reveals that this effect can be explained by the reaction of the separating ligands to form C_2_H_6_. This fragmentation pathway is confirmed and dynamically studied using irradiation-driven molecular dynamics^24^ simulations, which allow on-the-fly changes in (i) the molecular topology of the system, (ii) the atomic types and partial charges, and (iii) the interatomic interaction potentials for the parent ion and the molecular fragments. An important achievement of this study is the validation of the IDMD simulation results against the experiment. The present results partly explain the results of surface science experiments with the MeCpPtMe_3_ molecule,^10,13^ suggest a reinterpretation of earlier results on electron-induced fragmentation of the biologically relevant molecule deoxyribose,^48^ and pave the way for the theoretical treatment of dissociative processes accompanied by the formation of new molecules with the reactive rCHARMM force field.^33^

## Supporting Information Available

The evaluation of appearance energies and isotopic contributions to the peaks in the mass spectra, rCHARMM force field parameters, specification of the molecular topology, and simulation results for the localized energy transfer mechanism (PDF)
