# Supplementary material for: Release of Neutrals in Electron-Induced Ligand Separation from MeCpPtMe3: Theory Meets Experiment
Source: J Phys Chem A. 2025 Feb 13;129(8):2016–23. doi: 10.1021/acs.jpca.4c08259 (PMC11873943; doi:10.1021/acs.jpca.4c08259)
Supplement: Supplementary file 1 — jp4c08259_si_001.pdf [file jp4c08259_si_001.pdf]

Supporting Information for Release of Neutrals in Electron-Induced Ligand Separation from $MeCpPtMe_3$: Theory Meets Experiment

Hlib Lyshchuk,[†] Alexey V. Verkhovtsev,[*,¶] Jaroslav Kočišek,[†] Juraj Fedor,[*,†] and Andrey V. Solov'yov[¶]

†*J. Heyrovský Institute of Physical Chemistry, Czech Academy of Sciences, Dolejškova 3, 18223 Prague, Czech Republic*

‡*Department of Physical Chemistry, University of Chemistry and Technology, Technická 5, 16628 Prague, Czech Republic*

¶*MBN Research Center, Altenhöferallee 3, 60438 Frankfurt am Main, Germany*

E-mail: verkhovtsev@mbnexplorer.com; juraj.fedor@jh-inst.cas.cz

Evaluation of appearance energies for each mass-spectrum peak

We have analyzed each significant peak in the $\mathrm{MeCpPtMe}_{3-n}^{+}$ ($n = 0 - 3$) mass spectrum. The results are presented in Table S1. Three types of analyses have been performed:

- Platinum has five major isotopes with masses of 192, 194, 195, 196, and 198 amu. Thus, a mass peak with a given m/z has an uncertain number of H atoms, since this mass can be achieved by a combination of different Pt isotopes and the corresponding number of H atoms. A natural abundance of Pt and C isotopes has been assumed when fitting the contribution of various combinations to the cumulative mass spectrum shown in Fig. 1(a).

  The fitting procedure was as follows: let the experimentally measured intensity in the given group of mass peaks be $I(m)$ (with m being a discrete mass). Let $I_n(m)$ be a hypothetical intensity (as a discrete function of mass), which arises if the neutral fragments contain n H atoms (this means that the parent cation lost n H atoms). This $I_n(m)$ is directly given by the natural abundance of isotopes. We have assumed that the $I(m) = \sum_n c_n I_n(m)$ and the coefficients c_n have been varied in a least-squares fit procedure. These coefficients are the "contrib." column in Table S1.

  The results of such a fitting are the first four columns in Table S1. For example, the peak at $m/z = 270$ contains a 70% contribution from $C_6H_4Pt^+$ (corresponding to the loss of 3 carbon and 12 hydrogen atoms from the parent cation and a 29% contribution from $C_6H_6Pt^+$ (corresponding to the loss of 3 carbon and 10 hydrogen atoms from the parent cation. Only the channels contributing with more than 10% are shown in the table.

- From the list of removed atoms, possible neutral channels were hypothesized (fifth column in Table S1), and appearance energies (AEs) for the ionic species were calculated

using DFT at the B3LYP/LanL2DZ level of theory (sixth column). The AEs were obtained as differences of the sums of the electronic and zero-point energies of the products and reactants as

$$\mathrm{AE}_{\mathrm{DFT}} = \sum_{i}^{\text{products}} E_i - E[\mathrm{MeCpPtMe_3}]. \tag{S1}$$

Here, E_i is the sum of the electronic and zero-point energies of a given fragment, and the sum goes over all the charged and neutral fragments. Atomic mass of the Pt atom used in the calculations was 194.9648 amu.

- The last two columns in Table S1 show the experimental AEs obtained by fitting the generalized Wannier threshold law, Eq. (1), to the ion-yield curves. In some cases, a change in the slope of the ion yield indicated two thresholds; these were fitted with a superposition of two Wannier curves with appearance energies AE_1 and AE_2. The fitting procedure is demonstrated by four examples in Figure S1.

A closer look at Table S1 shows that the experimental AE values are usually higher than the calculated ones. This is not surprising since the calculated values represent relaxation into the lowest energy channels, which often require complex rearrangement reactions. Such channels are not necessarily present in the experiment, or there might be barriers on the reaction pathways. The disagreement typically occurs when the cation starts to lose H atoms, making it difficult to always "find" a methyl group and form a methane molecule. For the peaks where the loss of H atoms is less probable, the experimental AEs show good agreement with the calculated values.

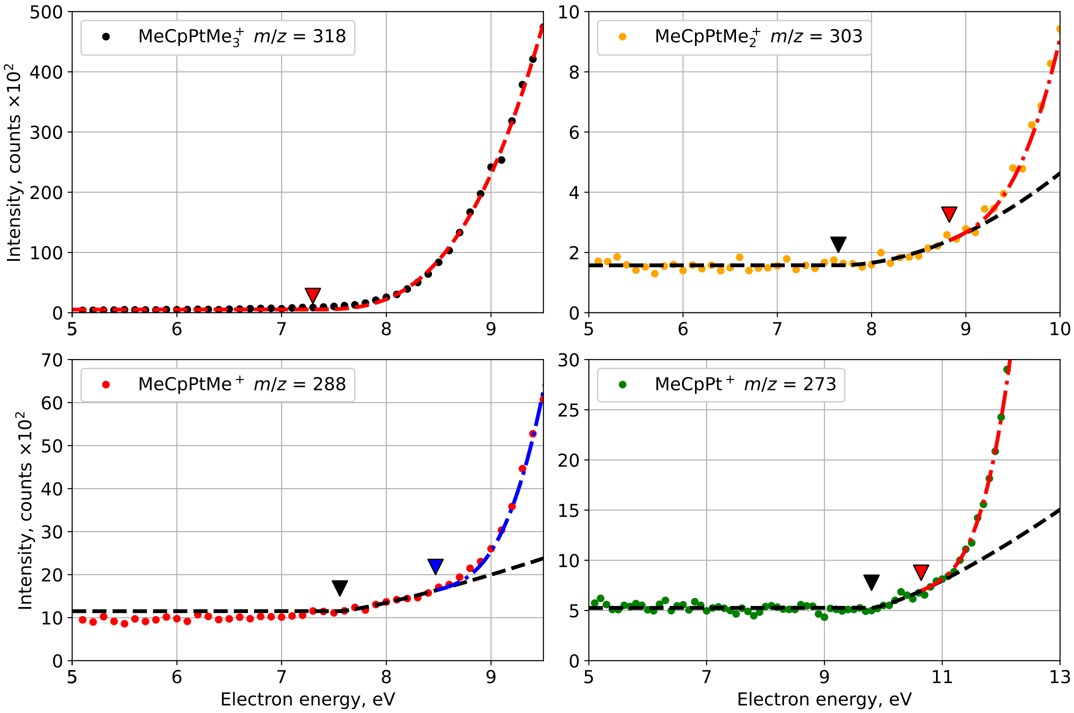

Figure S1: Example of the fitting procedure for four selected peaks in the mass spectrum. The dashed and dash-dotted curves represent the fitting curves, and the arrows indicate the appearance energies listed in the last two columns of Table S1.

Table S1: Appearance energies (AE) (in eV) for different fragments and the corresponding fragmentation channels. The column AE_{DFT} contains the AE values calculated by means of DFT at the B3LYP/LanL2DZ level of theory. The columns AE_1 and AE_2 contain the values obtained by fitting the experimental ion yield curves. The fragments marked with an asterisk contain one ^{13}C atom. Details are provided in the text.

m/z	Main frag.	Loss of	Contrib.	Neutral frag.	AE_{DFT}	AE_1	AE_2
**270**	$C_6H_4{}^{194}Pt^+$	3×C, 12×H	70%	$C_2H_6 + CH_4 + H_2$	10.32	12.16	12.85
	$C_6H_6{}^{192}Pt^+$	3×C, 10×H	29%	$C_2H_6 + CH_4$	8.22		
**271**	$C_6H_5{}^{194}Pt^+$	3×C, 11×H	78%	$C_2H_6 + CH_3 + H_2$	11.74	11.25	14.28
				$C_2H_6 + CH_4 + H$	11.84		
**272**	$C_6H_6{}^{194}Pt^+$	3×C, 10×H	72%	$C_2H_6 + CH_4$	8.22	10.03	11.24
	$C_6H_5{}^{195}Pt^+$	3×C, 11×H	24%	$C_2H_6 + CH_3 + H_2$	11.74		
				$C_2H_6 + CH_4 + H$	11.84		

Table S1 – continued

m/z	Main frag.	Loss of	Contrib.	Neutral frag.	AE_{G16}	AE_1	AE_2
**273**	$C_6H_6{}^{195}Pt^+$	3×C, 10×H	50%	C_2H_6 + CH_4	8.22	9.80	10.64
	$C_6H_7{}^{194}Pt^+$	3×C, 9×H	38%	C_2H_6 + CH_3	9.84		
**274**	$C_6H_6{}^{196}Pt^+$	3×C, 10×H	47%	C_2H_6 + CH_4	8.22	9.04	10.62
	$C_6H_7{}^{195}Pt^+$	3×C, 9×H	51%	C_2H_6 + CH_3	9.84		
**275**	$C_6H_7{}^{196}Pt^+$	3×C, 9×H	85%	C_2H_6 + CH_3	9.84	9.45	11.05
**276**	$C_6H_6{}^{198}Pt^+$	3×C, 10×H	82%	C_2H_6 + CH_4	8.22	9.58	–
	$C_6H_7{}^{196}Pt^+$(*)	3×C, 9×H	16%	C_2H_6 + CH_3	9.84		
**277**	$C_6H_7{}^{198}Pt^+$	3×C, 9×H	93%	C_2H_6 + CH_3	9.84	9.68	–
**278**	$C_6H_7{}^{198}Pt^+$(*)	3×C, 9×H	100%	C_2H_6 + CH_3	9.84	9.38	–
**285**	$C_7H_9{}^{192}Pt^+$	2×C, 7×H	10%	C_2H_6 + H	9.87	9.16	12.31
				CH_4 + CH_3	8.98		
	$C_7H_7{}^{194}Pt^+$	2×C, 9×H	90%	CH_4 + CH_3 + H_2	11.63		
				C_2H_6 + H_2 + H	12.52		
**286**	$C_7H_{10}{}^{192}Pt^+$	2×C, 6×H	34%	C_2H_6	7.36	9.32	12.73
				2×CH_3	10.91		
	$C_7H_8{}^{194}Pt^+$	2×C, 8×H	28%	2×CH_4	8.49		
				C_2H_6 + H_2	9.28		
	$C_7H_7{}^{195}Pt^+$	2×C, 9×H	38%	CH_4 + CH_3 + H_2	11.63		
				C_2H_6 + H_2 + H	12.52		
**287**	$C_7H_9{}^{194}Pt^+$	2×C, 7×H	71%	C_2H_6 + H	9.87	8.60	10.94
				CH_4 + CH_3	8.98		
	$C_7H_8{}^{195}Pt^+$	2×C, 8×H	14%	2×CH_4	8.49		
	$C_7H_7{}^{196}Pt^+$	2×C, 9×H	14%	CH_4 + CH_3 +H_2	11.63		
				C_2H_6 + H_2 + H	12.52		

Table S1 – continued

m/z	Main frag.	Loss of	Contrib.	Neutral frag.	AE_{G16}	AE_1	AE_2
**288**	$C_7H_{10}{}^{194}Pt^+$	2×C, 6×H	88%	C_2H_6	7.36	7.56	8.99
	$C_7H_9{}^{195}Pt^+$	2×C, 7×H	10%	CH_4 + CH_3	8.98		
				C_2H_6 + H	9.87		
**289**	$C_7H_{10}{}^{195}Pt^+$	2×C, 6×H	92%	C_2H_6	7.36	7.48	8.46
**290**	$C_7H_{10}{}^{196}Pt^+$	2×C, 6×H	99%	C_2H_6	7.36	7.22	8.24
**291**	$C_7H_{10}{}^{196}Pt^+$(*)	2×C, 6×H	88%	C_2H_6	7.36	7.22	8.59
	$C_7H_9{}^{198}Pt^+$	2×C, 7×H	28%	CH_4 + CH_3	8.98		
				C_2H_6 + H	9.87		
**292**	$C_7H_{10}{}^{198}Pt^+$	2×C, 6×H	99%	C_2H_6	7.36	7.74	–
**299**	$C_8H_9{}^{194}Pt^+$	1×C, 7×H	93%	CH_3 + 2×H_2	10.86	9.17	11.27
				CH_4 + H_2 + H	10.96		
**300**	$C_8H_9{}^{195}Pt^+$	1×C, 7×H	70%	CH_3 + 2×H_2	10.86	9.01	11.22
				CH_4 + H_2 + H	10.96		
	$C_8H_{10}{}^{194}Pt^+$	1×C, 6×H	28%	CH_4 + H_2	10.04		
**301**	$C_8H_{11}{}^{194}Pt^+$	1×C, 5×H	69%	CH_3 + H_2	10.39	9.13	10.50
				CH_4 + H	10.49		
	$C_8H_9{}^{196}Pt^+$	1×C, 7×H	19%	CH_3 + 2×H_2	10.86		
				CH_4 + H_2 + H	10.96		
**302**	$C_8H_{11}{}^{195}Pt^+$	1×C, 5×H	70%	CH_3 + H_2	10.39	7.76	10.28
				CH_4 + H	10.49		
	$C_8H_{12}{}^{194}Pt^+$	1×C, 4×H	21%	CH_4	7.97		

Table S1 – continued

m/z	Main frag.	Loss of	Contrib.	Neutral frag.	AE_{G16}	AE_1	AE_2
**303**	$C_8H_{13}{}^{194}Pt^+$	1×C, 3×H	39%	CH_3	8.65	7.65	8.82
	$C_8H_{12}{}^{195}Pt^+$	1×C, 4×H	17%	CH_4	7.97		
	$C_8H_{11}{}^{196}Pt^+$	1×C, 5×H	40%	$CH_3 + H_2$	10.39		
				$CH_4 + H$	10.49		
**304**	$C_8H_{13}{}^{195}Pt^+$	1×C, 3×H	71%	CH_3	8.65	7.65	9.09
	$C_8H_{12}{}^{196}Pt^+$	1×C, 4×H	25%	CH_4	7.97		
**305**	$C_8H_{13}{}^{196}Pt^+$	1×C, 3×H	75%	CH_3	8.65	8.64	10.05
	$C_8H_{11}{}^{198}Pt^+$	1×C, 5×H	22%	$CH_3 + H_2$	10.39		
				$CH_4 + H$	10.49		
**318**	$C_9H_{16}{}^{194}Pt^+$	–	100%	–	7.19	7.30	–
**319**	$C_9H_{16}{}^{195}Pt^+$	–	100%	–	7.19	7.37	–
**320**	$C_9H_{16}{}^{196}Pt^+$	–	100%	–	7.19	7.28	–
**321**	$C_9H_{16}{}^{196}Pt^+(^*)$	–	100%	–	7.19	7.30	–
**322**	$C_9H_{16}{}^{198}Pt^+$	–	100%	–	7.19	7.27	–

rCHARMM force field parameters

The interatomic interactions for $MeCpPtMe_3^+$ and its fragments were described using the rCHARMM force field,[1] see the Section “rCHARMM force field” in the main text. All the force field parameters were derived from the DFT calculations performed in this study.

Tables S2–S5 list the rCHARMM force field parameters used in the irradiation-driven molecular dynamics (IDMD)[2] simulations to describe the parent $MeCpPtMe_3^+$ ion and the molecular fragments.

Table S2 lists the parameters for the bonded interactions, Eq. (2). It includes information on the equilibrium bond lengths r_0, force constants k^r_{ij}, and dissociation energies D_e for different bonds of the parent $MeCpPtMe_3^+$ ion and the $MeCpPtMe_n^+$ ($n = 1, 2$) fragments.

Table S3 lists the parameters for the angular interactions, Eq. (3), for the $MeCpPtMe_3^+$ parent ion. It includes information on the equilibrium angles θ_0 and force constants k^θ_{ijk}.

In the optimized geometry, the $MeCpPtMe_3^+$ parent ion has two Pt–C bonds with a dissociation energy of ∼1.52 eV (bonds labeled as Pt–C_2 and Pt–C_4) and one bond (Pt–C_3) with a higher dissociation energy of 2.50 eV. After the loss of a methyl group, the types of the two carbon atoms bonded to the Pt atom change from $C_{2,4}$ (depending on whether the Pt–C_2 or Pt–C_4 bond broke first) and C_3 to C′. The dissociation energies of the two remaining Pt–C′ bonds increase up to 3.21 eV. After the loss of two methyl groups, the type of the last carbon atom bonded to the Pt atom changes from C′ to C″, and the dissociation energy of the Pt–C″ bond changes to 2.73 eV. The C′–H and C″–H interactions remain the same as in the parent ion and, therefore, are not repeated in the table.

Several additional force field parameters have been introduced to simulate the reaction path from the transition state to the endpoint corresponding to ethane formation (see Figure 3 and Figure 4(b,c)). This is a rather complex chemical transformation that requires the specification of additional interaction parameters to be simulated within the classical MD framework. For this purpose, an additional C_2–C_4 bonded interaction has been introduced,

Table S2: Parameters of the covalent bonded interaction, Eq. (2), for the $MeCpPtMe_3^+$ parent ion and $MeCpPtMe_n^+$ ($n = 1, 2$) fragments. The atomic notations refer to the geometry of $MeCpPtMe_3^+$ shown in Fig. 4.

bond type	r_0 (Å)	k_{ij}^r (kcal/mol Å^{-2})	D_{ij} (kcal/mol)	D_{ij} (eV)
**$MeCpPtMe_3^+$:**				
$C_0 - C_0$	1.446	326.7	116.0	5.03
$C_0 - H_0$	1.080	313.1	107.4	4.66
$C_0 - C_1$	1.490	417.9	110.1	4.77
$C_1 - H$	1.095	435.8	78.0	3.28
$C_{2,4} - H$	1.095	422.1	78.0	3.28
$C_3 - H$	1.095	422.1	115.3	5.00
$Pt - C_0$	2.592	116.1	50.0	2.17
$Pt - C_{2,4}$	2.068	215.5	35.0	1.52
$Pt - C_3$	2.099	158.8	57.6	2.50
**$MeCpPtMe_2^+$:**				
$Pt - C'$	2.054	215.5	74.0	3.21
**$MeCpPtMe^+$:**				
$Pt - C''$	2.041	215.5	63.0	2.73
**Transition state leading to ethane formation :**				
$C_2 - C_4$	1.700	326.7	200.0	8.67
$C_{ME} - C_{ME}$	1.446	326.7	113.7	4.93
$C_{ME} - H$	1.095	422.1	78.0	3.38

see Table S2. The cutoff distance for this interaction was set to 2.5 Å, which is smaller than the equilibrium C_2–C_4 distance of 2.9 Å in the optimized geometry of $MeCpPtMe_3^+$. During an MD simulation, the carbon atoms of the two methyl ligands may come closer than the specified cutoff distance due to the thermal vibrations of the atoms. When this happens, a covalent bond is formed between the C_2 and C_4 atoms without breaking the Pt–C_2 and Pt–C_4 bonds. This model is based on the potential energy surface calculated with DFT (see Figure 3), which shows that in the transition state, the carbon atoms of the two methyl groups are located close to the Pt atom.

To allow the formation of the C_2–C_4 bond, the valences of the C_2 and C_4 atoms have been increased to 5, which can be interpreted as the overlap of the electronic orbitals of the carbon atoms as they approach each other. When a Pt–C bond is broken, the released methyl radical acquires some kinetic energy and flies away from the Pt atom, making it

impossible to simulate the ethane formation process without the additional C_2–C_4 bonded interaction.

Once the metastable state decays and the Pt–C_2 and Pt–C_4 bonds are broken, the types of the carbon atoms change from $C_{2,4}$ to C_{ME}, which have the standard valence of 4. The equilibrium length of the C_{ME}–C_{ME} bond and its dissociation energy correspond to the values obtained for ethane in DFT calculations.[3]

The dissociation energy for the C_2–C_4 bond was set to a large value of ∼8.7 eV, which was chosen to ensure that the two methyl ligands come together at a distance corresponding to the C–C distance in the transition state, as determined by the DFT calculations. The difference between the interaction energies in the C_2–C_4 bond and in the C_{ME}–C_{ME} bond in ethane is approximately equal to the energy difference between the neutral ethane molecule and its singly charged anion, as calculated by DFT. Thus, the change in the interaction energy upon changing the atom types from $C_{2,4}$ to C_{ME} reflects the local redistribution of the electronic density in the $MeCpPtMe_3^+$ ion as it evolves from the transition state to the singly charged $MeCpPtMe^+$ fragment and a neutral C_2H_6 molecule.

Table S3: Parameters of angular interactions, Eq. (3), for $MeCpPtMe_3^+$ employed in this study. θ_0 denotes the equilibrium angle and k^{θ}_{ijk} is the force constant.

angle type	θ_0 (deg.)	k^{θ}_{ijk} (kcal/mol rad^{-2})
$C_0 - C_0 - C_0$	108.0	80.1
$C_0 - C_0 - C_1$	126.0	80.1
$C_0 - C_0 - H_0$	125.0	45.5
$C_0 - C_1 - H$	111.0	45.5
$H - C_1 - H$	108.0	37.1
$H - C_{MM} - H$	111.0	37.1
$H - C_{M1} - H$	111.0	37.1
$H_0 - C_0 - Pt$	120.0	80.1
$C_2 - Pt - C_3$	92.0	80.1
$C_3 - Pt - C_4$	86.5	80.1
$C_0 - Pt - C_{2,4}$	120.0	80.1
$C_0 - Pt - C_3$	120.0	80.1
$Pt - C_{2,4} - H$	108.0	80.1
$Pt - C_3 - H$	108.0	80.1
$Pt - C_0 - C_1$	120.0	80.1

Table S4 lists the parameters of the Lennard-Jones potential, Eq. (4), describing the van der Waals interaction between atoms of $MeCpPtMe_3^+$. Parameters for the Pt atom were taken from Ref. 4. Parameters for all other atoms were generated using the SwissParam web interface.[5]

Table S4: Parameters of the Lennard-Jones potential, Eq. (4), describing the van der Waals interaction between atoms of $MeCpPtMe_3^+$.

Atom	ε (kcal/mol)	$r^{\mathrm{min}}/2$ (Å)
C_1	0.055	2.175
$C_{2,4}$	0.055	2.175
C_3	0.055	2.175
C_0	0.068	2.090
H	0.022	1.320
Pt	7.800	1.423

Table S5: Partial atomic charges for the singly charged $MeCpPtMe_n^+$ ($n = 0 - 3$) species, employed in the IDMD simulations. The charge distributions were obtained through the natural bond orbital analysis using the Gaussian software.[6]

**$MeCpPtMe_3^+$:**		**$MeCpPtMe_2^+$:**		**$MeCpPtMe^+$:**		**$MeCpPt^+$:**	
Atom	Charge, $\|e\|$	Atom	Charge, $\|e\|$	Atom	Charge, $\|e\|$	Atom	Charge, $\|e\|$
C_0	0.2180	C_0	0.0881	C_0	0.2315	C_0	0.2604
C_0	-0.1095	C_0	-0.1624	C_0	-0.1193	C_0	-0.3747
C_0	-0.0503	C_0	-0.2429	C_0	-0.1101	C_0	-0.3103
C_0	-0.1833	C_0	-0.3130	C_0	-0.1664	C_0	-0.2754
C_0	0.0719	C_0	-0.2142	C_0	0.1114	C_0	0.0173
C_1	-0.3441	C_1	-0.6728	C_1	-0.3442	C_1	-0.6925
H	0.1265	H	0.2488	H	0.1275	H	0.2695
H	0.1534	H	0.2668	H	0.1495	H	0.3031
H	0.1315	H	0.2589	H	0.1341	H	0.2703
Pt	0.3685	Pt	0.6860	Pt	0.4625	Pt	0.4378
H_0	0.1344	H_0	0.2633	H_0	0.1310	H_0	0.2648
H_0	0.1281	H_0	0.2779	H_0	0.1231	H_0	0.2592
H_0	0.1319	H_0	0.2559	H_0	0.1377	H_0	0.2887
H_0	0.1329	H_0	0.2631	H_0	0.1388	H_0	0.2817
C_2	-0.3571	C'	-0.7501	C''	-0.3700		–
H	0.1224	H	0.2448	H	0.1227		–
H	0.1177	H	0.2451	H	0.1220		–
H	0.1256	H	0.2448	H	0.1182		–
C_3	-0.2968	C'	-0.7150		–		–
H	0.1174	H	0.2416		–		–
H	0.1188	H	0.2398		–		–
H	0.1228	H	0.2455		–		–
C_4	-0.3461		–		–		–
H	0.1266		–		–		–
H	0.1183		–		–		–
H	0.1205		–		–		–

Molecular topology for $MeCpPtMe_3^+$

The simulations employing the rCHARMM force field require the specification of the molecular topology for the parent molecular ion and its fragments. The $MeCpPtMe_3^+$ ion contains a methylcyclopentadienyl (MeCp) ligand bound to the Pt center. Cp is a η^5-ligand, meaning that all five of its carbon atoms are bound to the Pt atom due to the overlap of the π molecular orbitals of the C atoms with the electronic orbitals on the Pt atom.[7]

Several approaches have been proposed[8–10] to define the topology for metallocene organometallic complexes and their derivatives, such as $MeCpPtMe_3$. In this study, the Pt–Cp interaction was modeled by specifying five bonded interactions between the Pt atoms and the carbon atoms of the Cp ring[9] (see Figure 4(a)). The BDE for each Pt–C_0 bond was evaluated by calculating the Pt–Cp interaction energy using DFT and dividing it by five. The calculated value of the Pt–Cp interaction energy, 4.6 eV, is within the typical range of metal–Cp dissociation energies ($\sim 4-6$ eV) in different metallocenes.[11–14]

Figure S2 shows the relative abundance of the parent $MeCpPtMe_3^+$ ion and $MeCpPtMe_n^+$ ($n = 0-2$) fragments produced in the localized energy transfer mechanism when the energy is transferred locally to a specific covalent bond of the parent $MeCpPtMe_3^+$ ion: the weaker Pt–C_2 and Pt–C_4 bonds (panel (a)) and a stronger Pt–C_3 bond (panel (b)).

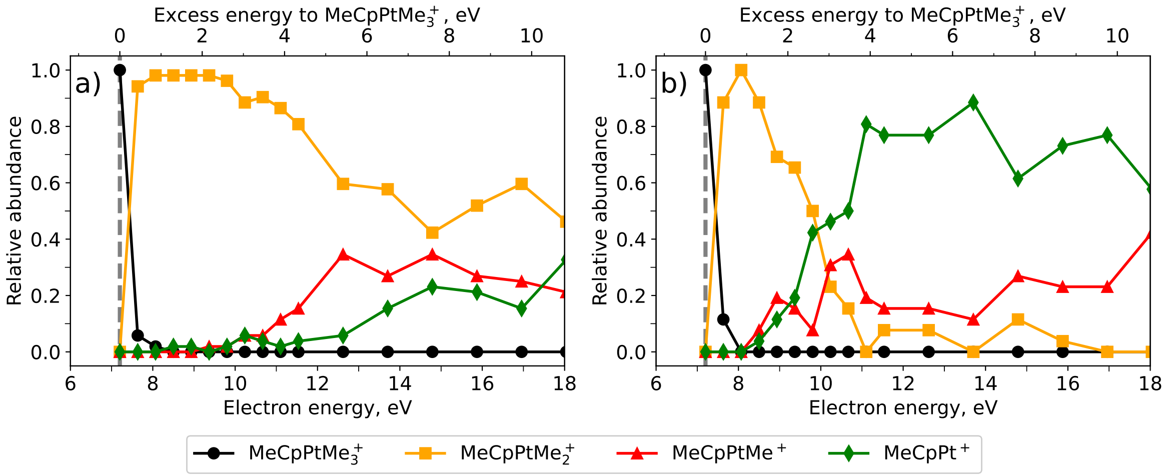

Figure S2: Relative abundance of the parent $MeCpPtMe_3^+$ ion and $MeCpPtMe_n^+$ ($n = 0-2$) fragments produced in the localized energy transfer mechanism when the energy is transferred locally to a specific covalent bond of the parent $MeCpPtMe_3^+$ ion: the weaker Pt–C_2 and Pt–C_4 bonds (a) and a stronger Pt–C_3 bond (b). The vertical dashed lines indicate the ionization energy of the neutral parent molecule $MeCpPtMe_3$ as determined by the DFT calculations.

Figure S3 shows a cumulative normalized mass spectrum of $MeCpPtMe_3$ for the fragment mass range $190 - 330$ amu in the electron energy range of 0 to 80 eV with an energy step size of 0.5 eV.

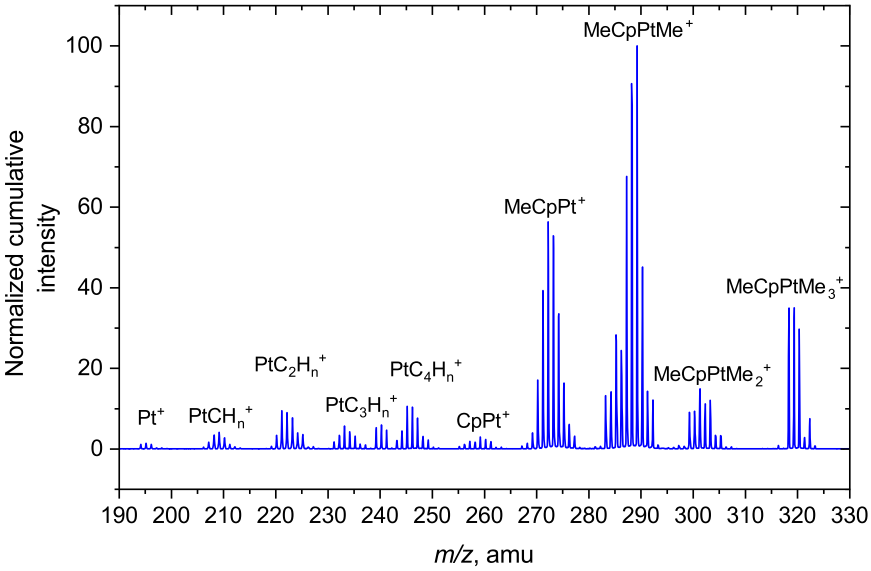

Figure S3: Cumulative normalized mass spectrum for the fragment masses $190 - 330$ amu in the electron energy range of 0 to 80 eV with an energy step size of 0.5 eV.

References

(1) Sushko, G. B.; Solov'yov, I. A.; Verkhovtsev, A. V.; Volkov, S. N.; Solov'yov, A. V. Studying Chemical Reactions in Biological Systems with MBN Explorer: Implementation of Molecular Mechanics with Dynamical Topology. *Eur. Phys. J. D* **2016**, *70*, 12.

(2) Sushko, G. B.; Solov'yov, I. A.; Solov'yov, A. V. Molecular Dynamics for Irradiation Driven Chemistry: Application to the FEBID Process. *Eur. Phys. J. D* **2016**, *70*, 217.

(3) Tamukong, P. K.; Khait, Y. G.; Hoffmann, M. R. Accurate Dissociation of Chemical Bonds Using DFT-in-DFT Embedding Theory with External Orbital Orthogonality. *J. Phys. Chem. A* **2017**, *121*, 256–264.

(4) Heinz, H.; Vaia, R. A.; Farmer, B. L.; Naik, R. R. Accurate Simulation of Surfaces and Interfaces of Face-Centered Cubic Metals Using 12–6 and 9–6 Lennard-Jones Potentials. *J. Phys. Chem. C* **2008**, *112*, 17281–17290.

(5) Zoete, V.; Cuendet, M. A.; Grosdidier, A.; Michielin, O. SwissParam, A Fast Force Field Generation Tool for Small Organic Molecules. *J. Comput. Chem.* **2011**, *32*, 2359–2368.

(6) Frisch, M. J.; Trucks, G. W.; Schlegel, H. B.; Scuseria, G. E.; Robb, M. A.; Cheeseman, J. R.; Scalmani, G.; Barone, V.; Petersson, G. A.; Nakatsuji, H. et al. Gaussian 16 ES64L-G16RevC.01. 2016; Gaussian Inc. Wallingford CT.

(7) Elschenbroich, C. *Organometallics*, 3rd ed.; Wiley-VCH: Weinheim, 2006.

(8) Doman, T. N.; Landis, C. R.; Bosnich, B. Molecular Mechanics Force Fields for Linear Metallocenes. *J. Am. Chem. Soc.* **1992**, *114*, 7264–7272.

(9) Timofeeva, T. V.; Lii, J.-H.; Allinger, N. L. Molecular Mechanics Explanation of the Metallocene Bent Sandwich Structure. *J. Am. Chem. Soc.* **1995**, *117*, 7452–7459.

(10) de Hatten, X.; Cournia, Z.; Huc, I.; Smith, J. C.; Metzler-Nolte, N. Force-Field Development and Molecular Dynamics Simulations of Ferrocene–Peptide Conjugates as a Scaffold for Hydrogenase Mimics. *Chem. Eur. J.* **2007**, *13*, 8139–8152.

(11) Lewis, K. E.; Smith, G. P. Bond Dissociation Energies in Ferrocene. *J. Am. Chem. Soc.* **1986**, *106*, 4650–4651.

(12) Révész, A.; Szepes, L.; Baer, T.; Sztáray, B. Binding Energies and Isomerization in Metallocene Ions from Threshold Photoelectron Photoion Coincidence Spectroscopy. *J. Am. Chem. Soc.* **2010**, *132*, 17795–17803.

(13) Rowland, T. G.; Sztáray, B.; Armentrout, P. B. Metal–Cyclopentadienyl Bond Energies in Metallocene Cations Measured Using Threshold Collision-Induced Dissociation Mass Spectrometry. *J. Phys. Chem. A* **2013**, *117*, 1299–1309.

(14) Thorman, R. M.; Matsuda, S. J.; McElwee-White, L.; Fairbrother, D. H. Identifying and Rationalizing the Differing Surface Reactions of Low-Energy Electrons and Ions with an Organometallic Precursor. *J. Phys. Chem. Lett.* **2020**, *11*, 2006–2013.
